# Trigeminal Pontine Sign: From Imaging to Diseases Beyond Trigeminal Neuralgia

**DOI:** 10.3390/diseases12120327

**Published:** 2024-12-12

**Authors:** Marialuisa Zedde, Rosario Pascarella

**Affiliations:** 1 Neurology Unit, Stroke Unit, Azienda Unità Sanitaria Locale-IRCCS di Reggio Emilia, Viale Risorgimento 80, 42123 Reggio Emilia, Italy; 2 Neuroradiology Unit, Azienda Unità Sanitaria Locale-IRCCS di Reggio Emilia, Viale Risorgimento 80, 42123 Reggio Emilia, Italy; pascarella.rosario@ausl.re.it

**Keywords:** trigeminal nerve, trigeminal pontine sign, MRI, multiple sclerosis, VZV, NMOSD

## Abstract

The so-called trigeminal pontine sign has been described as a marker of different diseases, from multiple sclerosis to herpetic infections. First, it has been proposed as linear hyperintensity in the pons on the Magnetic Resonance Imaging (MRI) of patients with multiple sclerosis and trigeminal neuralgia. After these descriptions, it has been reported as incidental findings in the same patients and in patients with HSV or VZV infections. In addition, patients with neuromyelitis optica spectrum disorders (NMOSD) have been more rarely described with this neuroradiological sign. In this review, the main anatomical and neuroradiological issues underlying the trigeminal pontine sign are described, together with the limitations of the published studies from the clinical and neuroimaging point of view. Finally, the association with different diseases is detailed.

## 1. Introduction

The trigeminal nerve (TN) or cranial nerve V is the largest cranial nerve, with an extensive distribution territory [1]. As a mixed sensory and motor nerve, it transmits sensory afferent fibers from the face and mouth and provides motor innervation to the muscles of mastication. The TN is susceptible to a wide range of diseases, including vascular, ischemic, inflammatory, and neoplastic conditions. Magnetic resonance imaging (MRI) is the primary modality for evaluating patients with symptoms related to TN dysfunction. The TN originates in the brainstem and follows a long, complex course toward its distribution areas. Anatomically, the nerve can be divided into four segments: brainstem, cisternal, Meckel’s cave–cavernous sinus, and extracranial ones. Trigeminal neuralgia is a type of neuropathic pain that manifests in the sensory distribution of the TN. It is defined by episodes of intense, sharp, and lancinating pain, which occur in a stereotypical pattern and can last from a fraction of a second to up to two minutes. The condition, classified as classical trigeminal neuralgia, has a prevalence of approximately 1–2 cases per 10,000 individuals [2]. While its exact etiology remains uncertain, multiple hypotheses have been proposed. The most widely accepted explanation attributes the condition to neurovascular conflict at the cisternal segment of the trigeminal nerve [3]. The pathophysiology underlying the vascular contact as a cause of facial pain in trigeminal neuralgia remains a subject of debate. However, it is widely accepted that vascular compression at the root entry zone (REZ) of the trigeminal nerve leads to focal demyelination. This demyelination disrupts normal nerve conduction, causing “cross-talk” between adjacent nerve fibers. The resulting aberrant signal transmission alters pain-processing mechanisms, ultimately manifesting as neuropathic pain [4].

Independent from the presence of symptoms, including trigeminal neuralgia, the so-called trigeminal pontine sign, as linear pontine hyperintensity on T2W MRI starting from the REZ and extending towards the tegmentum, has been described in the last 25 years as associated with several conditions: mainly symptomatic for trigeminal neuralgia but not exclusively associated with symptoms. First described in patients with multiple sclerosis [5,6,7] as a linear pontine trigeminal root lesion, it was recently proposed as a marker of post-herpetic etiology of trigeminal neuralgia [8] and described in other demyelinating diseases as neuromyelitis optica spectrum disorders (NMOSDs) [9]. However, it remains poorly characterized, and it is frequently found in asymptomatic or never-symptomatic patients and in patients without demyelinating diseases. The term “trigeminal sign” is a recent proposal, but, as previously said, the association of trigeminal neuralgia with a solitary pontine lesion on MRI has been previously reported in isolated case series and case reports [10,11].

The main aim of this review is to summarize the available information about the trigeminal pontine sign and its MRI features, the potential meanings and associated diseases.

## 2. TN: Neuroanatomical Issues

The TN’s afferent fibers converge from its three primary sensory distributions within the Meckel cave. From there, the nerve passes through the porous trigeminus, traverses the cistern, and enters the pons. Along the cisternal segment, the myelin sheath undergoes a key transition: it shifts from being formed by Schwann cells (peripheral myelin) to oligodendrocytes (central myelin). This transitional region, often referred to as the transition zone, is a significant anatomical feature. The point where the nerve enters the pons is known as the root entry point. The root entry zone (REZ) of the trigeminal nerve is widely defined as the proximal cisternal segment where centrally myelinated fibers transition to peripherally myelinated fibers [12]. Central myelination, derived from oligodendrocytes, contrasts with peripheral myelination, which originates from Schwann cells. This structural difference makes the REZ more vulnerable to external compression, leading to hemifacial pain in the trigeminal nerve distribution. In contrast, the distal segments of the nerve are less susceptible to such compression. Previous studies have noted that the medial portion of the trigeminal nerve root has shorter central myelination compared to the lateral portion [13,14]. Variability exists in the length of the REZ, which typically measures between 3 and 7 mm, as well as in the length of the cisternal segment [14]. Peker et al. [14] conducted a detailed histological study of trigeminal nerve microanatomy, analyzing 100 nerves from 50 cadavers. Using Luxol fast blue staining (Sigma-Aldrich, St. Louis, MI, United States), they examined the length and morphology of the centrally myelinated segment. Their findings revealed variability: in some nerves, central myelin constituted less than a quarter of the cisternal length, while the maximum observed centrally myelinated segment reached 48% of the cisternal portion [14]. As noted by Peker [14], the term REZ refers specifically to the anatomical junction where a nerve meets the brainstem [15,16]. Historically, some researchers have conflated the REZ with the central-peripheral myelin transition zone (TZ), treating them as the same structure. However, Sindou et al. [17] argued that these terms describe distinct entities and should not be used interchangeably. Our detailed examination of trigeminal nerve specimens supports this distinction, confirming that the REZ and TZ are separate structures. Research on the extent of central myelin within the cranial nerve V and the structure of the TZ has been limited and very old. In 1931, Skinner [18] described aspects of the TZ in humans, dogs, cats, and rabbits, estimating that central myelin extended 3 mm in humans, though the number of specimens studied was not reported. Tarlov [19], in 1937, measured a length of 2.2 mm but similarly omitted sample size details. In the 1980s, Lang [20] analyzed 10 trigeminal nerves, finding an average central myelin length of 3.57 mm (range: 2–6 mm). Ziyal et al. [21] later observed a significantly longer length of 8–10 mm in six specimens. Although various authors have reported the length of this zone, few conducted systematic anatomical studies [13,17,22,23,24]. Peker et al. [14] analyzed 100 trigeminal nerves from 50 cadavers, representing the most comprehensive anatomical study of this nerve to date. The findings reveal that the central-peripheral myelin TZ lies, on average, 1.13 mm from the REZ on the medial aspect of the nerve and 2.47 mm on the lateral aspect. These results conclusively demonstrate that the REZ and TZ are anatomically and functionally distinct. Consequently, the terms “REZ” and “TZ” should not be used interchangeably.

Quantifying the length of the centrally myelinated segment as a fraction of the entire cisternal length is particularly valuable in clinical imaging. The cisternal portion of the nerve is well visualized using steady-state free precession (SSFP) MRI [25]. The cisternal segment can be differentiated into proximal (posterior) and distal (anterior) halves to assess vascular compression. Blood vessels impinging on the proximal (central myelin) portion are more likely to cause clinically significant symptoms compared to compression in the distal (peripheral myelin) segment. This distinction is crucial in evaluating potential causes of trigeminal nerve-related symptoms. As previously pointed out, the REZ refers to the cisternal portion of the trigeminal nerve as it enters the pons. This area is clinically significant, as it can be affected by various pathological processes, including vascular compression, tumors, and infections. In fact, vascular compression at the REZ is considered the most common cause of idiopathic trigeminal neuralgia. Primary tumors within the prepontine cistern that can involve the root entry zone include meningiomas, trigeminal schwannomas, epidermoid cysts, vestibular schwannomas, and lipomas [26]. Secondary neoplasms affecting this area may result from perineural tumors, spreading from head and neck malignancies, hematogenous metastases, or leptomeningeal tumor dissemination [27]. Additionally, inflammatory or infectious conditions can impact the root entry zone. These include sarcoidosis, viral encephalitis, herpes neuritis [28], and Lyme disease [26].

The trigeminal ganglion divides into multiple rootlets that traverse the prepontine cistern posteriorly to reach the brainstem. Within the brainstem, these fibers spread out to innervate three distinct sensory nuclei:

1. Principal sensory nucleus: located in the pontine tegmentum, this nucleus processes pressure and light touch sensations from all three divisions of the trigeminal nerve (V1–V3) [29,30].
2. Mesencephalic nucleus: situated at the pons-midbrain junction, it primarily receives proprioceptive input from the mandibular division (V3).
3. Spinal trigeminal nucleus: extending from the ponto-medullary junction to the upper cervical cord, this nucleus mediates pain and temperature sensations from V1–V3.

Second-order neurons originating from these nuclei project to the thalamus, while third-order neurons extend from the thalamus to the cerebral cortex, completing the sensory pathway [30].

The motor component of the trigeminal nerve originates in the motor nucleus, located in the floor of the fourth ventricle [31]. Upon exiting the brainstem, it travels medially to the sensory fibers within the prepontine cistern and bypasses the trigeminal ganglion to enter Meckel’s cave. At the skull base, it joins the sensory fibers of the mandibular division (V3), forming the mandibular nerve [32]. This motor branch is responsible for innervating the muscles of mastication, as well as the tensor tympani and tensor veli palatini muscles [29]. Structurally, the motor nucleus of the trigeminal nerve forms an oval column, situated anteromedially to the motor root and the principal sensory nucleus within the pons. The principal sensory nucleus lies lateral to the entering trigeminal root, while the mesencephalic nucleus forms a narrow column near the lateral edge of the central gray matter, positioned anterior to the upper fourth ventricle and cerebral aqueduct. Proprioceptive signals from the teeth, hard palate, and temporomandibular joint are transmitted through afferent fibers from the mesencephalic nucleus. These fibers descend in the mesencephalic tract, a crescent-shaped structure, to the motor nucleus, playing a key role in regulating mastication and bite force.

The spinal trigeminal tract, which originates in the sensory root of the pons, extends downward into the upper cervical spinal cord, where it terminates in the spinal trigeminal nucleus. This nucleus connects rostrally to the principal sensory nucleus. The cervical extension of the spinal tract accounts for instances where patients with upper cervical disk herniations experience trigeminal sensory neuropathy [33].

Several conditions may affect the brainstem and upper cervical cord, leading to symptoms involving the TN. Common lesions include multiple sclerosis and infarction. Less frequent causes include metastases, cavernous hemangiomas [34], hemorrhage, and arteriovenous malformations [26]. Rarely, rhombencephalitis may result from the retrograde extension of herpes simplex virus type 1 from the trigeminal ganglion into the brainstem [28].

In conclusion, the TN’s complex anatomy and extensive connections make it susceptible to a wide range of disorders, but a more limited range of diseases can affect the intra-axial segment with a trigeminal pontine sign on MRI.

## 3. Trigeminal Pontine Segment: Neuroradiological Issues

Conventional MRI can delineate five key anatomical components of the trigeminal nuclear complex and nerve within the brainstem [35]. These include the following:

- The sensory nucleus (touch and position sensation);
- The motor nucleus (muscle control for mastication);
- The mesencephalic nucleus (proprioception);
- The spinal nucleus and tract (pain and temperature sensation);
- The intrapontine fascicular part, the central pathway of trigeminal nerve fibers within the brainstem.

Despite advancements, most studies on MRI characteristics of brainstem demyelinating lesions in patients with trigeminal nerve symptoms have not analyzed the spatial relationship of these lesions to the primary anatomical components of the nerve [5,7,36]. Addressing this gap, Swinnen et al. [37] studied 43 patients (62.8% female) with demyelinating disease and trigeminal nerve symptoms. A notable limitation of their study was the use of multiple MRI systems over a broad time span, ranging from 1.0 T and 1.5 T scanners (e.g., Siemens Somatom, Siemens Sonata, Philips Achieva) to more advanced 3.0 T scanners (e.g., Philips Achieva). The imaging protocols varied:

1.0 T and 1.5 T scanners: Axial and coronal 4 mm proton density and T2-weighted images, along with high-resolution Gadolinium-enhanced T1-weighted images;

3.0 T scanners: Selective 8 mm axial FLAIR images or 3 mm multi-echo Fast Field Echo (m-FFE) images.

The study found unilateral lesions in 79% of patients. Of these,

51% had linear plaques involving the intrapontine fascicular part (81.8% of these patients had relapsing–remitting multiple sclerosis [RRMS]).

53.4% had lesions in the spinal nucleus and tract (69.5% had definite RRMS).

**Figure 1:**
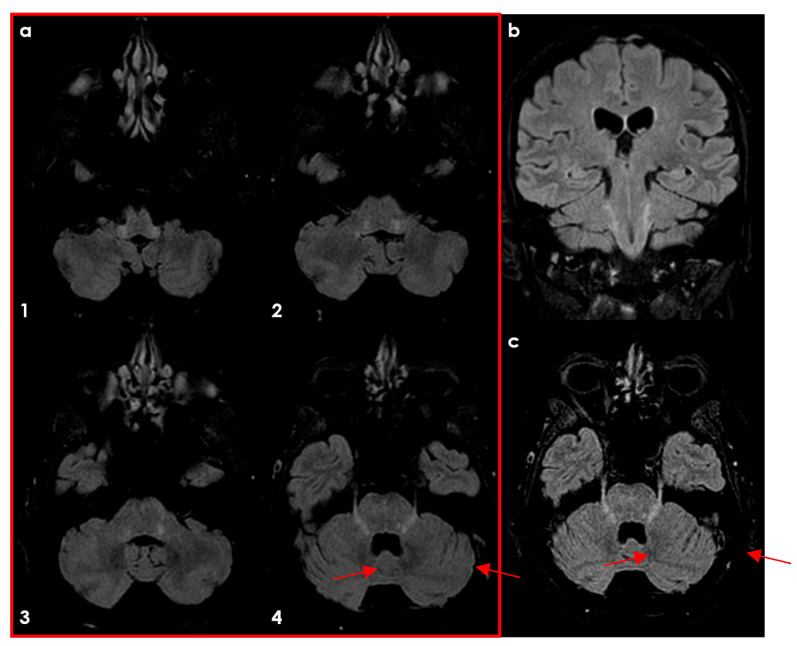
Axial FLAIR MRI in a patient with multiple sclerosis and trigeminal symptoms on the right side, showing, in ascending slices, panel (**a1**–**a4**), hyperintense signal in both trigeminal tract and a bilateral trigeminal pontine sign (red arrows). In panel (**b**), the coronal FLAIR is proposed, showing the longitudinal extension of pontine hyperintensities. In the panel (**c**), the axial oblique FLAIR slice, focused on the plane of cisternal TN, is reconstructed, showing a bilateral hyperintensity of both TN and intrapontine segment (red arrows).

**Figure 2:**
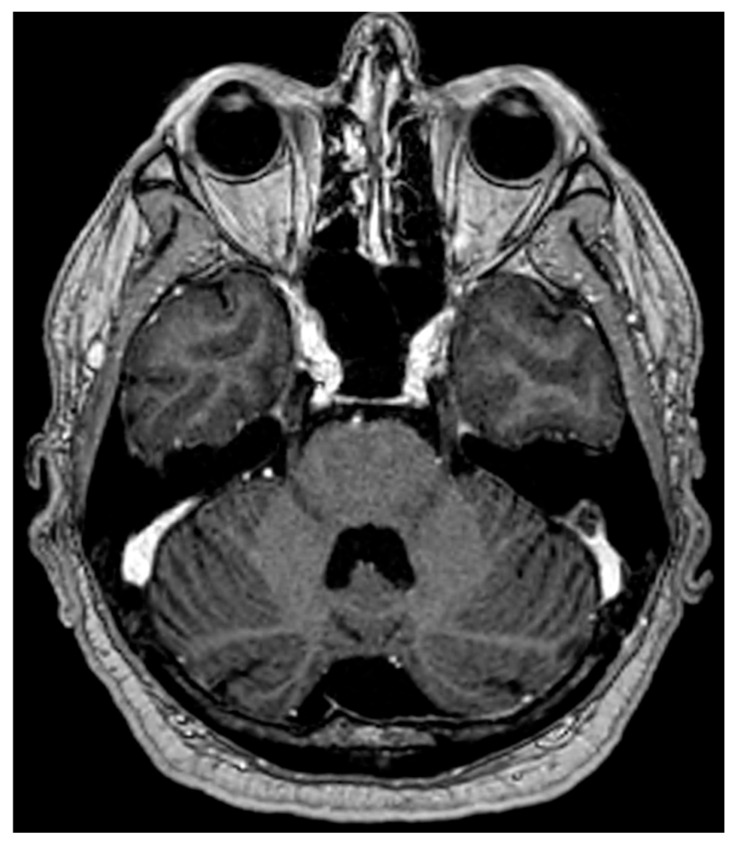
In the same patient shown in Figure 1, a post-contrast T1W MRI is proposed, focused on the plane of the cisternal TN (same plane as in Figure 1c), without contrast enhancement.

**Figure 3:**
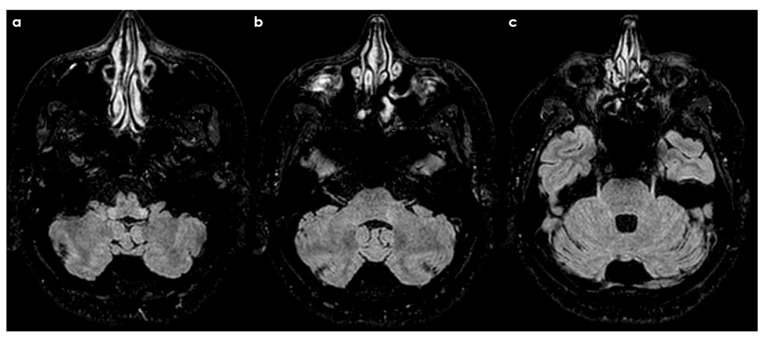
Three-month follow-up MRI of the same patients as in Figure 1 and Figure 2, after steroid treatment, showing a strong reduction in pontine hyperintensities with persisting hyperintense trigeminal tract in ascending axial FLAIR slices from (**a**–**c**).

**Figure 4:**
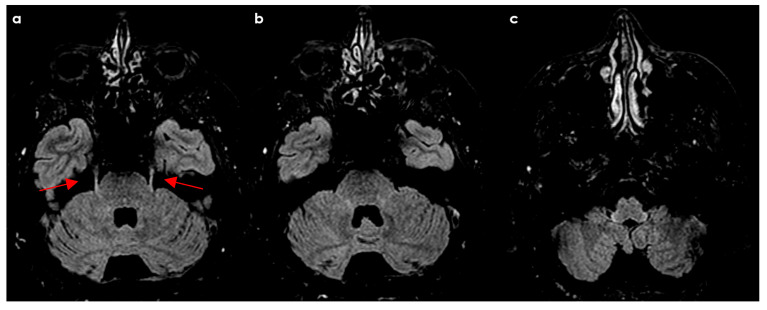
Six-month follow-up MRI of the same patients as in Figure 1, Figure 2 and Figure 3, showing further improvement of the findings with persisting linear hyperintensity along the TN course (red arrows) in ascending axial FLAIR slices from (**a**–**c**).

Lesions also appeared in the mesencephalic nucleus (20.9%), primary sensory nucleus (32.5%), and motor nucleus (11.6%). Among RRMS patients, 62% had lesions spanning multiple brainstem regions, with 81% involving the intrapontine fascicular part and 76% affecting the spinal nucleus and tract. However, the study noted that lesion characteristics are not specific. Similar features have been described in HSV-1–infected animals and patients with systemic inflammatory or infectious diseases [38,39,40]. Figure 1, Figure 2, Figure 3 and Figure 4 illustrate examples of trigeminal pontine signs in patients with demyelinating disease.

In MS patients, trigeminal lesions predominantly localize along the cisternal portion of the trigeminal nerve, often extending to the REZ and the pontine-medullary nucleus [5,41]. In a study of 1628 MS patients, 26 out of 28 individuals with the TN were found to have plaques at the REZ [7]. These lesions exhibit a distinct linear shape, with their long axis parallel to the trigeminal nerve, raising questions about the mechanisms driving lesion development in this specific region [5,6]. Histological studies have confirmed focal demyelination at the REZ in MS-related TN cases [4]. The REZ serves as a critical transition zone where central myelin (produced by oligodendrocytes) transitions to peripheral myelin (produced by Schwann cells). This region is particularly vulnerable to neurovascular compression, which may further contribute to lesion formation. Notably, 88.9% of abnormal signals detected in the cisternal portion of the trigeminal nerve are associated with the REZ or pontine lesions, suggesting that cisternal nerve involvement may often be secondary [4]. The trigeminal pontine sign refers to a linear MRI lesion observed at the intramedullary trigeminal root in the pons. This lesion is characterized by hyperintensity on T2WI and hypointensity on T1WI, aligning with the distinct features of REZ plaques [5,7].

Regarding the MRI technique, the main studies reporting as their objective the characterization of trigeminal neuralgia in patients with multiple sclerosis had differences that were also affected by the timeframe of enrollment [42]. In fact, in a systematic revision published in 2014 [42] and including 38 studies, the most relevant finding was the underreporting of imaging methodology. Only 39% of the studies reported the field strength of the employed scanner, being 1 T in two studies [41,43], 1.5 T in nine studies [5,36,40,44,45,46,47,48,49], and 3 T in two studies [50,51]. Moreover, two studies employed scanners of varying strengths: 0.6, 1.5, and 3 T [52], and 0.5 and 1.5 T [53]. Only 37% of the included studies detailed the MRI protocols, but only 64% of these last ones described all the sequences, and only 29% described all the sequence parameters of all the sequences. Despite these limitations, six out of fourteen studies described unilateral or bilateral lesions in the REZ of multiple sclerosis patients with trigeminal neuralgia or trigeminal autonomic cephalalgias (TACs) [5,40,46,53,54,55,56]. In a recent study [57] using 7 T MRI for assessing the prevalence of TN lesions in MS, the sequences used for this task were T1-MPRAGE, T2-FLAIR and fluid and white-matter suppression (FLAWS) based on the magnetization-prepared 2 rapid acquisition (MP2RAGE) sequence (FLAWS-MP2RAGE). FLAIR* and T2*-weighted imaging were utilized to detect central vein signs (CVSs) in trigeminal lesions [58]. For analysis, the affected trigeminal nerve was segmented into three distinct regions: the cisternal segment, the REZ, and the nuclear zone, based on anatomical landmarks and observed signal abnormalities. Studies have shown variability in the prevalence of trigeminal nerve (TN) lesions in MS, depending on the imaging techniques and study designs. Reported prevalence ranges from 1.7% in a large prospective study of 1628 patients using 3 T MRI to 23% in a smaller prospective study of 47 patients, also using 3 T MRI [7,59]. Although radiological findings are nonspecific in many cranial nerve pathologies, magnetic resonance imaging (MRI) with heavily T2-weighted three-dimensional (3D) steady-state free precession (SSFP) sequences, which have high spatial resolution and provide good contrast between cerebrospinal fluid and the cisternal segments of the cranial nerves, and contrast-enhanced T1-weighted sequences, facilitates the depiction of abnormalities [60].

In patients with trigeminal neuralgia, advanced neuroimaging techniques, such as diffusion tensor imaging (DTI), have identified white-matter microstructural abnormalities at the trigeminal REZ. These abnormalities are characterized by lower fractional anisotropy (FA) and higher mean diffusivity (MD), radial diffusivity (RD), and axial diffusivity (AD) [11,40,61,62]. FA is particularly sensitive to overall white-matter microstructural changes, while MD, RD, and AD provide more specific insights into neuroinflammation, myelination, and axonal integrity, respectively. Additionally, trigeminal neuralgia associated with solitary pontine lesions has been proposed as a distinct clinical entity in patients without multiple sclerosis. The largest study on this condition, published by Tohyama et al. [11], included 24 patients who presented with single brainstem lesions along the trigeminal nerve pathway, with no other supratentorial or infratentorial lesions. Long term after the removal of compression, the loss of FA persisted, but ADC normalized in the affected nerves, suggesting an improvement in conduction sensitivity and the reduction in edema in the trigeminal root after surgery. The re-establishment of diffusion could well be the reason for pain relief after surgery. Furthermore, from a practical standpoint, DTI metrics could be an effective biomarker for the confirmation of aggressiveness of a potential neurovascular compression found on imaging and could become an additional diagnostic tool for ascertaining its compressive behavior [63,64,65,66,67].

## 4. Trigeminal Pontine Sign: Definition and Etiologies

Trigeminal neuralgia and sensory disturbance are common in multiple sclerosis, and frequent trigeminal root entry zone (REZ) involvement has been reported [5,7,37,41]. Although REZ involvement on MRI is regarded to be a distinctive MRI finding in multiple sclerosis [37], it has rarely been investigated in NMOSDs in order to assess if a different rate of involvement is present between multiple sclerosis and NMOSDs. In a single study [9], the brain MRI of 128 consecutive multiple sclerosis patients and 46 NMOSD patients was evaluated in Japan, finding trigeminal REZ abnormality in 8.6% of the multiple sclerosis patients and 4.3% of the NMOSD patients. In the above reported study, of all patients with trigeminal REZ abnormality on MRI, three multiple sclerosis patients and one NMOSD patient presented with trigeminal sensory disturbance, and none with trigeminal neuralgia. Interestingly, in a study conducted by Swinnen et al. [37], centered on patients with demyelinating disease and trigeminal symptoms, 48.8% of patients had typical trigeminal neuralgia at the time of MRI, 27.9% presented with central sensible loss, 4.6% presented with an atypical neuralgic pain and abnormal findings on sensory examination, and 18.6% of patients were totally asymptomatic (MRI lesions were considered an incidental finding).

Several MRI studies have highlighted abnormalities in the REZ in multiple sclerosis patients, particularly in those experiencing trigeminal nerve-related symptoms. Early studies, which utilized low-field-strength MRI and thicker slice imaging, reported a prevalence of trigeminal REZ lesions ranging between 3% and 7% [5,41]. In contrast, more recent research employing high-resolution 3D MRI at 3 T with thin slices (1 mm) identified a significantly higher prevalence of 23% [7]. Sugiyama et al. [9], using a 1.5 T MRI system with 4 mm slice thickness, found a prevalence of 8.6%, suggesting that thinner slices and higher field strength improve the detection of trigeminal REZ abnormalities. The main information from the study conducted by Sugiyama et al. [9] is the lack of association between trigeminal neuralgia and trigeminal REZ abnormalities on MRI, although the retrospective design might have affected this issue and some transient abnormalities could have been missed without acute phase MRI in the case of trigeminal symptoms. A recent study on 120 Chinese multiple sclerosis patients employed 7 T MRI in order to assess the prevalence of lesional TN involvement [57]. In their study, the authors identified 45 trigeminal lesions in 19 out of 120 patients (15.8%), with 11 of the 19 patients (57.9%) showing bilateral involvement. The linear lesions extended along the trigeminal nerve, from the root entry zone (REZ; 57.8%, 26/45) to the pontine-medullary nucleus (42.2%, 19/45). Among the REZ lesions, 26.9% (7/26) exhibited the characteristic central venous sign. Based on these findings, the authors proposed an inflammatory demyelination mechanism underlying trigeminal nerve involvement in MS. Notably, only 10% of patients with detectable trigeminal abnormalities had clinical trigeminal neuralgia.

In NMO patients, the dorsal medulla is commonly affected, correlating with areas of high aquaporin-4 (AQP4) expression [68,69,70]. Although the trigeminal REZ is not a typical brainstem lesion site in NMOSD, it may still be affected due to the AQP4 expression in transitional zones of the central and peripheral nervous systems. This makes the trigeminal REZ a potential target for anti-AQP4 antibodies in NMOSD [71]. In fact, in a multicenter study of brainstem manifestations in NMOSD, the prevalence of trigeminal neuralgia was 2.5% [70], similar to the 2.2% prevalence of REZ hyperintense signal in Sugiyama et al. [9]. However, no NMOSD patients with trigeminal neuralgia in this cohort showed trigeminal REZ abnormalities on MRI [9]. While pontine trigeminal REZ abnormalities are observed in both multiple sclerosis and NMOSD, they are not disease-specific and cannot differentiate between these conditions. However, the role of trigeminal REZ abnormalities in NMOSD and their potential link to trigeminal neuralgia have to be clarified.

Trigeminal neuralgia is highly prevalent in patients with multiple sclerosis (2.1%) [72]. The incidence of trigeminal neuralgia in MS patients was found to be significantly higher at 149 per 100,000 person-years (95% CI: 108–190) compared to 9.9 per 100,000 person-years (95% CI: 9.5–10.3) in the general outpatient population. Among MS patients with available MRI data (*n* = 41), a demyelinating lesion near the trigeminal ganglia was observed in 63% of cases. However, in 12% of these patients, the lesion was contralateral to the TN symptoms. Notably, the median time between TN onset and MRI acquisition was 2.5 years (range: 0.1–18.4 years, *n* = 40). The detection rate of demyelinating lesions was higher with 3 T MRI compared to 1.5 T MRI, with 82% (9/11) of cases identified on 3 T imaging versus 57% on 1.5 T. Additionally, a prior study using 3 T MRI revealed central trigeminal involvement in 23% of MS patients, though this did not correlate with facial sensory symptoms [7].

**Figure 5:**
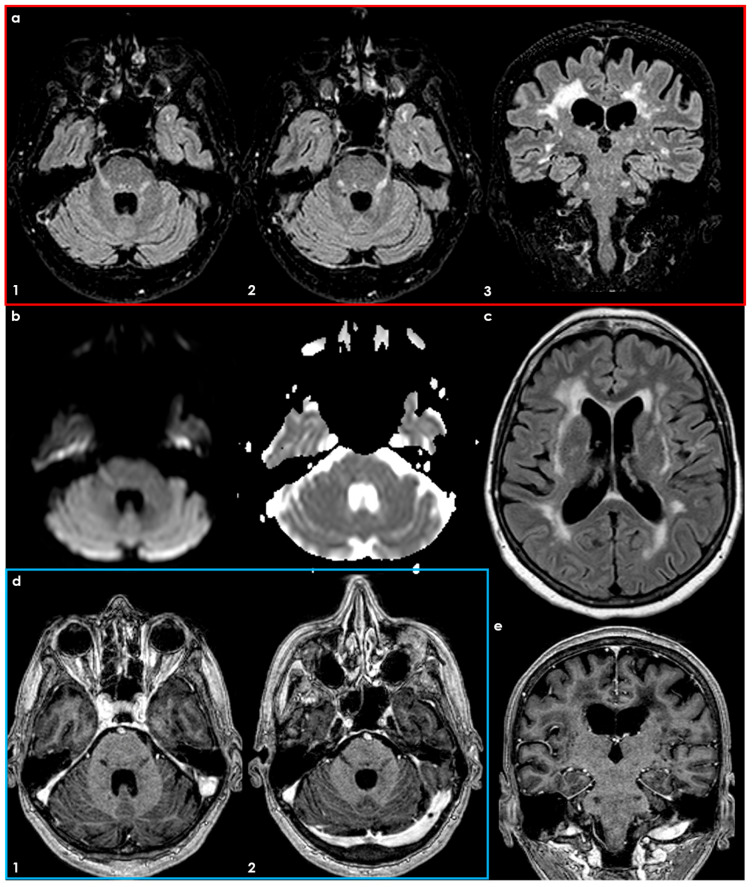
Patient with VZV recurrent infections: Axial panel (**a1**,**a2**) and coronal panel (**a3**) FLAIR MRI showing a bilateral trigeminal pontine sign. In panel (**b**), DWI and ADC slices at the same level. In panel (**c**), a supratentorial small vessel disease pattern is detailed with white-matter hyperintensities in the supratentorial location. In panels (**d**,**e**), the axial and coronal post-contrast T1W-MRI shows the lack of contrast enhancement of the pontine lesion.

More recently, the trigeminal pontine sign has been proposed as highly suggestive for a herpetic etiology in patients with trigeminal neuralgia [8], excluding from the analysis patients with multiple sclerosis and proposing a series of seven patients with a clinical diagnosis of herpes zoster (HZ) (four with CSF confirmation of the diagnosis). In 6/7 of these patients, a T2 hyperintense linear pontine sign was described, using 1.5 T brain MRI; only 1/7 patients had nerve atrophy. These lesions had a linear appearance, involving the pathway from the trigeminal REZ toward the dorso-lateral area of the pons, where the trigeminal tract and the nucleus are located. However, in previously reported cases, the location of the MRI abnormal signal was not the REZ and the isolated pons, but a continuously long high-signal lesion corresponding to the right spinal trigeminal nucleus and tract, extending from the lower pons to the second cervical segment of the spinal cord [73] or a non-linear pontine lesion [74,75]. In addition, in the ten patients with cranial herpes zoster and brainstem lesions described by Haanpaa, only one had a lesion in the REZ but was not isolated [76,77]. However, these conclusions need confirmatory studies. HZ is an acute, localized infection caused by the varicella-zoster virus (VZV), a neurotropic alpha-herpesvirus. Following a prior chickenpox infection, the VZV persists in a latent form within the dorsal root ganglia. Reactivation triggers viral replication, leading to its spread down the sensory nerve to the skin or mucosa, resulting in unilateral vesicular eruptions accompanied by sharp, radiating pain confined to a dermatome [77]. HZ typically begins with localized prodromal pain, followed by the appearance of erythematous patches, vesicle formation, rupture, and crusting. While lesions generally heal within 7–10 days, complete recovery may take longer. In oral mucosa, lesions do not crust due to the wet environment but follow a similar progression [77]. The intense pain associated with HZ is primarily caused by damage to neural and epithelial cells from the VZV, along with a strong inflammatory reaction. This damage to the nervous system can make individuals more susceptible to postherpetic neuralgia (PHN), a chronic pain condition that remains in the affected area after HZ lesions have healed. PHN is marked by severe burning pain, allodynia, and hyperalgesia. The duration of PHN symptoms can range from weeks to months; though, in some cases, they may continue for years or even reappear long after the initial HZ outbreak. PHN occurs due to several factors: (I) direct damage to nerves from VZV, affecting dorsal root ganglia and peripheral nerves during the acute phase of HZ; (II) inflammatory response, where intense inflammation worsens nerve and skin damage, amplifying pain; and (III) sensitization, where both peripheral and central nervous system changes lead to increased nerve sensitivity, impaired pain control, and the widening of pain response areas. An example of a VZV-related trigeminal sign is illustrated in Figure 5.

Some isolated pathological reports of patients with this finding showed demyelinating changes in the pons at the REZ [46]. Postmortem brain examinations in patients with trigeminal neuralgia and multiple sclerosis are uncommon. When such studies have been conducted, demyelination is typically observed in the pons, particularly at the REZ of the TN [78,79,80,81].

While neurovascular compression is the primary cause of “idiopathic” trigeminal neuralgia in most cases, its role in MS-related trigeminal neuralgia remains controversial. The neurovascular relationships of the trigeminal nerve have not been fully investigated in MS patients, and many authors have dismissed vascular compression as a potential factor. Notably, Brisman and Jannetta contraindicated microvascular decompression (MVD) in MS-related TN cases [80,82,83]. However, isolated reports challenge this perspective. Lazar and Kirkpatrick documented a case where an MS patient with trigeminal neuralgia had both a demyelinating plaque at the REZ and vascular compression of the nerve observed during surgery [84]. It is important to note that demyelination is a nonspecific response to injury and has also been observed at the REZ in patients with trigeminal neuralgia unrelated to MS [85]. Postmortem studies have revealed demyelinating plaques bilaterally affecting the REZ in MS patients [86]. Further insights come from experimental models, particularly studies of herpes simplex virus type 1 (HSV-1)–induced demyelination. In an animal model, HSV-1 infection via the cornea resulted in demyelination at the trigeminal REZ. Double immunoperoxidase staining revealed that astrocytes, identified using glial fibrillary acidic protein (GFAP), were the primary infected cells. By day 6 of post-infection, GFAP staining was absent in the inferior medial REZ, despite intact myelin indicated by myelin basic protein (MBP) staining. Between days 8 and 14, the progressive loss of MBP staining followed the earlier astrocyte depletion, suggesting a unique demyelination sequence distinct from other demyelinating conditions like multiple sclerosis or progressive multifocal leukoencephalopathy [87]. The demyelinated lesions observed by Nakashima et al. [5] closely resembled those seen in HSV-1 animal models, characterized by similar shape, size, and localization. It is well established that HSV-1, following experimental corneal inoculation, spreads transaxonally to the central nervous system via the ophthalmic branch of the trigeminal nerve, leading to selective demyelination of the intramedullary trigeminal root [87,88,89]. The similarities in lesion localization and morphology between HSV infections and multiple sclerosis suggest the possibility of a shared underlying pathogenic mechanism. If this is the case, it becomes crucial to determine whether HSV infection actively contributes to the formation of these distinctive MS lesions or if the inflammatory processes in MS reactivate latent HSV. In one of five MS patients with a history of recurrent trigeminal neuralgia, elevated anti-HSV antibody levels were found in both serum and the CSF during an episode of trigeminal neuralgia. However, HSV-specific DNA was not detected in the CSF by the polymerase chain reaction [5]. This finding raises the possibility that the trigeminal root may have sustained prior damage from herpes infection, with the increase in anti-HSV antibodies potentially reflecting a nonspecific activation of HSV-specific B cells during an multiple sclerosis relapse, rather than a new herpetic outbreak. It is well established that HSV remains dormant in the trigeminal ganglia, and HSV DNA is frequently detected in these ganglia in more than half of healthy controls, as well as in the brains of individuals with multiple sclerosis [90,91,92].

## 5. Conclusions

The trigeminal pontine sign, defined as axial linear T2W-MRI hyperintensity from the REZ towards the tegmentum, has been described in several diseases, such as inflammatory and infectious. In particular, multiple sclerosis and NMOSD have been associated with this finding independently from symptoms of TN involvement. VZV infection has been considered as a cause, but this sign is far from being pathognomonic of herpetic infections in the central nervous system. The incidental identification of a trigeminal sign in MRI investigations should trigger some questions for clarifying the history of the patients and, in some cases, could allow for the proposal of a diagnostic pathway to exclude some inflammatory or post-infectious conditions.
